# Evaluation of Current and Future Medical Staff Knowledge on the Course of Trauma Patient Management

**DOI:** 10.7759/cureus.64132

**Published:** 2024-07-09

**Authors:** Anna Dąbrowska, Wiktoria Malik, Dorota Czachor, Weronika Jarych, Anna Wściślak, Zuzanna Świąder, Łucja Komisarczyk, Piotr Pałczyński

**Affiliations:** 1 Student Scientific Circle of Emergency Medicine, Medical University of Gdansk, Gdansk, POL; 2 Department of Emergency Medicine, Medical University of Gdansk, Gdansk, POL

**Keywords:** management of trauma patient, trauma course, emergency medicine, education, trauma patient

## Abstract

Introduction: Management of injuries, especially in life-threatening situations, is critical to morbidity and mortality for trauma patients. The qualifications of medical staff and students in medicine, emergency medical services, and nursing help to ensure consistent, high-quality care for patients. The study aimed to assess the knowledge of our staff and learners in the management of trauma patients.

Material and methods: The study was carried out using a proprietary research tool consisting of 47 questions, including six independent variables. The knowledge assessment tool has been divided into five categories according to the degree of difficulty. The questions have been created based on the current guidelines of the ERC 2021, ITLS, PTLS, and TCCC.

Results: The study included 295 subjects (medical students, nursing students, students of emergency medical services, physicians, paramedics, and nurses). The vast majority of respondents (79.7%) have never participated in a certified trauma course. Respondents could obtain a total of 117 points for answering all questions. The highest score was 111 points, and the lowest was 26 points. The average score was 63 points. Paramedics received the highest average score of 78 points. The question with the smallest number of correct answers concerned the priority procedure in the case of an electric shock victim.

Conclusion: Better training in trauma patient management is needed for both current medical staff and students. A certified trauma course is a good source of knowledge and skills, but it would need to be repeated periodically. This would ensure an increase in the competence of medical staff involved in the care of trauma patients.

## Introduction

Injuries are one of the most common causes of death (47% from age 1 to 46) [1-2]. The constant development of industry and technology causes their continuous growth [3]. In Poland, injuries are the third-most common cause of death. There are many causes of injuries, from traffic and work accidents, falls, and assaults [3] to injuries related to military conflicts [4]. The latter should also be remembered, especially due to the current situation beyond our eastern border in Ukraine. Knowledge of current medical guidelines is crucial, especially when it comes to life-threatening conditions. Therefore, guidelines are updated every few years to ensure that patients receive the best possible care. Good post-traumatic care is important because it can reduce post-injury mortality by up to 30% [5]. Many causes of death can be prevented within the first few hours [5], especially since a significant peak in deaths occurs within the first hour of the event [2]. It is necessary that both practicing staff and those in training are familiarized with evidence-based medicine-centered recommendations. There is no doubt that practical skills are crucial, especially in stressful situations that require decisive action, but they do not exist without an appropriate theoretical background. The literature has shown that education in the management of trauma patients is insufficient not only among students [2,6-7] but sometimes also among doctors [6]. Appropriate management of trauma patients is significant as most of them are young and of working age, and injuries often have physical, emotional, and socio-economic consequences [8]. Trauma is one of the leading public health concerns and demands the appropriate knowledge and expertise to deliver timely care. The study aimed to assess the knowledge of healthcare professionals and students to identify strengths and weaknesses in their knowledge levels and, in the future, perhaps identify areas that require improvement in training programs. This study was presented as a meeting poster at the Central European Emergency Medicine Congress “CEEM 2023" on May 12, 2023.

## Materials and methods

The aim of the study was to evaluate the knowledge of current and future medical staff in the management of trauma patients. We wanted to enable respondents to test their knowledge and encourage them to improve their skills.

The study was conducted using a proprietary research tool consisting of 47 questions, including six independent variables. The 41 questions testing knowledge has been divided into five categories according to the degree of difficulty: I-very basic (four), II-basic (14), III-intermediate (10), IV-advanced (10), and V-highly specialized (three). The questions have been created based on the current guidelines of the European Resuscitation Council (ERC 2021), International Trauma Life Support (ITLS), Prehospital Trauma Life Support (PHTLS), and Tactical Combat Casualty Care (TCCC).

The survey was conducted between November 24, 2022, and March 5, 2023. We reached our study group through social media. The form was also distributed by employees of healthcare institutions and by e-mail. The study included adults who are studying medicine, emergency medical services, or nursing, or are graduates of the above-mentioned fields. Participation in the anonymous study was voluntary, and respondents could resign from participation at any time during the questionnaire. The research project and the research tool were approved by the independent Bioethics Committee for Research at the Medical University.

The original article was performed according to the SQUIRE-EDU (Standards for QUality Improvement Reporting Excellence in Education) Guidelines [9].

We used Statistica v.13.3 for statistical analysis. Pearson’s chi-squared test, the Mann-Whitney U test, and the Kruskal-Wallis non-parametric test were applied to analyze the relationships between variables. We assumed a statistical significance level of p < 0.05.

## Results

**Figure 1:**
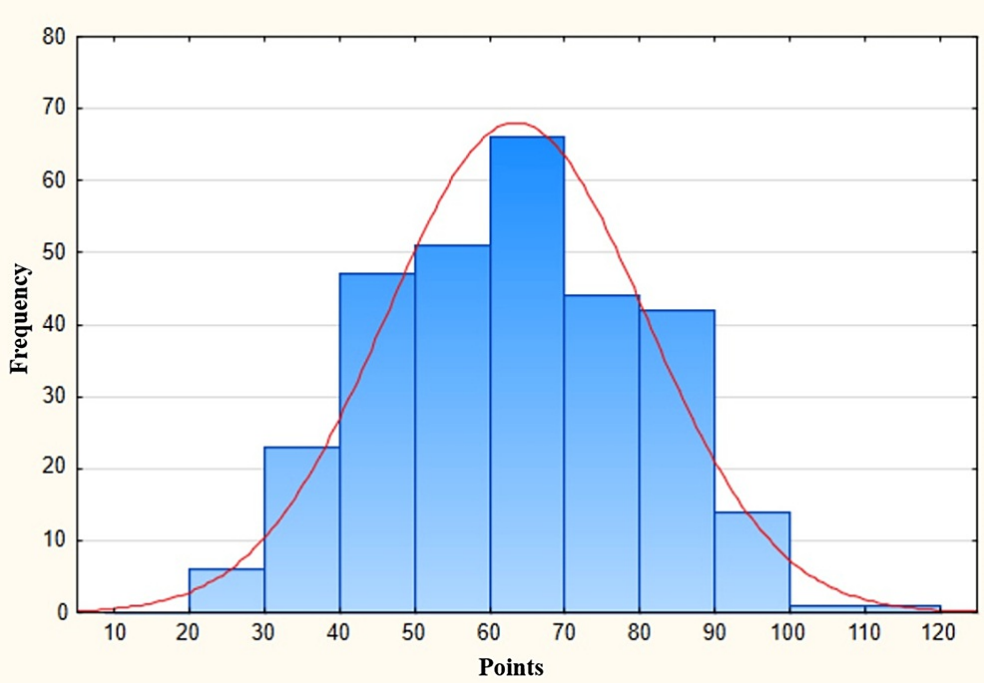
Normal distribution of the obtained results

The questionnaire was filled out by 295 respondents who met the inclusion criteria for the study, and their answers were analyzed. The majority of study participants were women (56.9%), people under 25 years of age (59.3%), and students (60%). More than half of respondents work or study in cities with a population over 250k (66.8%). Participation in a certified trauma course over the past year was declared by only 7.8% of the respondents, and the vast majority (79.7%) had never participated in such a course. Respondents could obtain a total of 117 points for answering all questions. The highest score was 111 points (obtained by an emergency medicine specialist), and the lowest was 26 points. The average score was 63 points. Figure 1 shows the normal distribution of the obtained results (Chi2 = 14.305, df = 8, p = 0.074). 

Then we performed a comparative analysis in the following groups: first, medical students before emergency medicine classes (N = 75) with medical students after emergency medicine classes (N = 31); second, medical students after emergency medicine classes (N = 31) with paramedic students (N = 46); third, doctors (N = 42) with paramedics (N = 39) and nurses (N = 35); and the last one, health care workers not participating in a certified trauma course (N = 71) with people who participated in such a course (N = 45).

Medical students before emergency medicine classes scored an average of 57 points, while students after such classes scored 62 points. The most difficult question among medical students was about the priority procedure for an electrically shocked victim (category IV: advanced level). Only 5.66% correctly indicated cervical spine stabilization. The Mann-Whitney U test showed no statistically significant differences between the results of these students (U = 905.5; p = 0.075). Most medical students were also unfamiliar with the decompression site for tension pneumothorax (category IV: advanced question), with only 34.91% providing the correct answer. Medical students after emergency medicine classes (90.32%) more often gave the correct answer compared to students before emergency medicine classes (70.67%) (Chi2 = 4.702, df = 1, p = 0.03).

Students of emergency medical services obtained an average higher score (71 points) than medical students after emergency medicine classes (62 points). The Mann-Whitney U test showed that the difference in results was statistically significant in the compared groups (U = 460; p = 0.009). Statistically significant differences were observed between the levels of first aid knowledge (category I: very basic level). Students of emergency medical services (95.65%) more frequently indicated the proper management for rescuing a drowned person compared to medical students after emergency medicine classes (80.65%) (Chi2 = 4.48, df = 1, p = 0.034). A statistically significant difference was also observed in the level of knowledge regarding the management of mass accidents (category III: intermediate level). Medical students after emergency medicine classes (96.77%) answered correctly more often compared to students of emergency medical services (73.91%) (Chi2 = 6.897, df = 1, p = 0.009).

Among healthcare workers, the highest average score was achieved by paramedics (78 points), followed by doctors (73 points) and nurses (57 points). The Kruskal-Wallis test was performed to assess whether there were significant differences in the level of knowledge between the above-mentioned three groups. To facilitate analysis, the questions were divided into five groups according to difficulty level. Respondents could get a maximum of four points for questions from category I (very basic level). The analysis of these questions showed significant differences (H = 14.164, p = 0.001) in the level of knowledge of paramedics (4 points) and nurses (3 points ± 1). For questions in category II (basic level), a maximum of 14 points could be obtained. Significant differences (H = 23.378, p < 0.001) in the level of knowledge between doctors (10 points ± 1) and paramedics (12 points ± 1) were observed, as well as between paramedics and nurses (10 points ± 2.5). Questions in category III (intermediate level) allowed for a score of 10 points. There were significant differences (H = 18.337, p = 0.001) in the knowledge level between doctors (6 points ± 1.5) and paramedics (8 points ± 1.5), as well as between paramedics and nurses (5 points ± 1.5). For questions in category IV (advanced level), it was also possible to score 10 points. The analysis of these questions revealed significant differences (H = 21.653, p < 0.001) in the knowledge level between doctors (5 points ± 1.5) and paramedics (6 points ± 1.5), as well as paramedics and nurses (4 points ± 1.5). The questionnaire included three questions in category V (highly specialized), the analysis of which showed significant differences (H = 12.46, p = 0.002) in the knowledge level between doctors (1.5 points ± 0.5) and nurses (1 point ± 0.5).

People who have ever participated in a certified trauma course (77 points) scored on average more points compared to respondents who have never participated in such a course (65 points). Participation in a certified trauma course was declared by 46% of paramedics, 40.5% of doctors, and 28.6% of nurses. The Mann-Whitney U test showed that the difference in results in these groups was statistically significant (U = 940; p < 0.001). The most difficult question again concerned priority management in the case of an electric shock victim. Only 9.48% of respondents in this group answered correctly. In turn, as many as 74.14% of those surveyed were not familiar with the components of the pediatric triangle. Statistically, significant differences in the level of knowledge in this group of respondents were observed. People who participated in a certified trauma course (60%) more often chose the correct procedure in the case of CPR with a foreign body impaled in the precardiac area compared to respondents who have never participated in such a course (35.21%) (Chi2 = 6.843, df = 1, p = 0.009). In the question about the management of persistent bleeding after the application of a tactical tourniquet, only 6.67% of respondents who had ever attended the course gave an incorrect answer. Incorrect answers were more common among people who did not participate in the course (23.94%) (Chi2 = 5.762, df = 1, p = 0.016). 

## Discussion

This survey is the first research in the Polish medical education area that checks the level of knowledge of trauma patient management among specialists and students of current and future medical staff. The results indicate insufficient knowledge of current and future medical staff in the management of trauma patients, which seems to be a crucial skill regarding the proper implementation of healthcare tasks nowadays [10]. The results obtained in this research clearly indicate that the level of knowledge among current and future medical staff in this area is insufficient. The average score in the survey was 63 out of 117 points, which is unsatisfactory considering the fact that the study was conducted among people directly related to this field-students and graduates of selected medical faculties (medicine, nursing, emergency medical services).

The study was conducted at a time when, after years of relative political stability, new threats were emerging. In the context of the ongoing war in Ukraine, we should reflect on whether Polish healthcare providers will be competent enough to take care of trauma patients in case of an escalation of the military conflict to NATO countries. Moreover, despite the intense fight against terrorism, there is still a high level of terrorist attacks in Europe [11]. Therefore, it seems obvious that healthcare professionals should be qualified to effectively treat potential victims, who are mainly trauma patients and may require specific treatment for, e.g., blast injuries [12].

With this study, we would also like to initiate a discussion on the quality of specialist medical care in the presented area as well as in the wider context of emergency medicine in Poland. Compared to the organizational culture of systems in the United States or Western Europe, where researchers, through evaluation, data collection, and analysis, thoroughly look for the causes of system imperfections on many levels [13], Polish reality presents itself as an area requiring extensive reforms. An example may be a tendency to base research on the quality of the system's quality only on the points at which patients reach a certain stage of treatment. Instead, we should focus on creating exponents for assessing the effectiveness of a treatment [14].

The study showed that we should focus more on the education of students of medicine, nursing, and emergency medical services in the field of emergency medicine, which would require changes in the curriculum. For example, medical students in Poland have only 120 hours of emergency medicine classes during their six years of study, of which only a small percentage involve the topic of the management of trauma patients [15]. Another example is that nursing students in Poland, during their three-year bachelor's studies, have only 45 hours of classes on the basics of emergency medicine [16]. According to the results of this study, emergency medicine classes have a positive impact on the knowledge of medical students in some areas, such as the management of persistent bleeding after the application of a tactical tourniquet or pain treatment.

Emergency medical services students generally demonstrate a better understanding of some topics than medical students, particularly in rescuing a drowned person, in priority management in the case of bilateral traumatic amputation, or in fluid therapy. It suggests that the Emergency Medical Services curriculum may be more focused on or effective in these spheres. More and more doctors and paramedics involved in emergency and battlefield medicine emphasize the need for better education of both medics and laymen in this field [17]. It was shown that students of emergency medical services more often repeat and focus on knowledge in the field of emergency medicine. The absence of frequent repetition of this specialized knowledge by medical students results in a diminished understanding of certain areas and a tendency to forget it [18].

However, there are some areas in which both medical and emergency medical services students show a significant lack of knowledge. These areas include priority actions for an electrocution victim and the assessment of the pediatric triangle in a pediatric trauma patient. This indicates the need for further development and improvement of study programs to better cover these topics. Zargaran et al. suggest that emergency medicine simulation training can result in significant increases in both competency and confidence among medical students. Emergency medicine simulation training may be an invaluable mechanism for the delivery of teaching in the context of reduced patient-facing teaching opportunities [19].

The results of this study also show that respondents who completed a certified trauma course are generally more likely to correctly answer questions about guidelines and trauma management [20] than those who have never attended one. This trend is noticeable both in the context of specific ITLS guidelines and in knowledge of the practical aspects of trauma management. These findings recommend that certified trauma courses can significantly contribute to improving competencies in the field of trauma medicine, as there is a need for regular repetition and updating of knowledge to maintain a high level of skills [21]. The aim of emergency training is not only to improve knowledge but also to acquire practical skills, which translate into more effective action in life-threatening situations [8]. On the other hand, there are some areas where the differences between the groups are not so marked. For example, responses to questions about electrocution, first aid, or fluid therapy for a burn patient showed no significant differences between those who completed the trauma course and those who did not. Our results are consistent with some meta-analyses that show there is no evidence from controlled trials that ATLS or similar programs impact outcomes for trauma victims. Although there is some evidence that educational initiatives improve hospital and pre-hospital staff's knowledge of available emergency interventions [22,23].

Healthcare workers who are not directly related to trauma medicine should also have some basic knowledge about the management of trauma patients. In a life-threatening situation, early application of critical interventions plays a key role, often at the level of a primary facility hospital. Therefore, optimizing the process increases the chances of saving the patient's life [24].

Putting aside situations related to warfare and terrorism, it should also be mentioned that external causes of death, including trauma, are responsible for as much as 50% of deaths among people aged 0 to 19 [25]. Therefore, as traumatic injuries typically impact young people, having knowledge and skills in the management of such patients is of great importance [26]. Moreover, it should be noted that mortality in traumatic cardiac arrest reaches 96.2% [27]. These alarming statistics lead us to reflect on how important it is to educate medical staff in the field of treating trauma patients.

It may be assumed that recent changes in guidelines in these areas may not have been properly considered or interpreted by healthcare workers, suggesting a lack of current updating of knowledge.

Emergency medicine guidelines are constantly changing and need to be reviewed and updated regularly. Even experienced professionals have fields of knowledge that require additional training and skill improvement. It appears that doctors, nurses, and paramedics working within the State Medical Rescue System should pay more attention to the constant updating of their skills. For example, participating in certified trauma courses, which are a good way to acquire knowledge in this area, as shown in this paper [28]. Recently, it was presented that trauma resuscitation training affects nurses' knowledge improvement, highlighting the need for training trauma care professionals to provide appropriate care [29]. Lam et al. proved that participating in training courses is an independent factor affecting knowledge level [30].

Therefore, the results of the study show the necessity for further improvement of the education of current and future medical staff in the field of emergency medicine to better prepare them for effective performance in crises.

Obviously, as with any other research, this study has its limitations. First, the survey was voluntary and relatively long (47 questions), which raises the suspicion that a large part of the respondents who spent time filling it in diligently were interested in the topic of emergency medicine and the management of trauma patients. Therefore, the results do not concern the entire population of current and future medical staff. However, we believe that this should not impact the general conclusions of the work, as expanding the group of respondents to include people not interested in emergency medicine should not change the trends shown in the paper. It can be assumed that the shortage of theoretical knowledge and knowledge of guidelines in the management of trauma patients is present in the entire population of future and current medical staff. Moreover, the study checked only theoretical knowledge, in particular the understanding of current guidelines. Practical skills were not assessed, so it is not possible to determine on this basis what the current competencies of the respondents are in the field of the management of trauma patients. Nevertheless, the normal distribution of the results shows that the survey itself was constructed correctly. Another limitation is the fact that the survey was conducted using only social media and e-mail, without the possibility of monitoring the respondents while they were filling out the questionnaire, so there is a probability that they used assistance. Finally, the relatively small number of respondents compared to the population of currently studying and working medical staff in Poland may distort the picture of theoretical knowledge and knowledge of the guidelines in this group. We assume that the findings of the study are preliminary and that further, thorough research is indispensable.

## Conclusions

In conclusion, the study underscored the importance of following current guidelines and continuous retraining, probably not only in the context of managing a trauma patient. Regarding the worrying results of the survey conducted, it would be advisable to repeat the survey on a wider group of respondents from future and current medical cadres to relate the results to the entire target study group. Further research is needed to establish the level of knowledge and monitor it to set up a remediation plan, implement it, and evaluate it regularly. Future research should consider the latest guidelines in trauma patient care, a qualitative assessment of theoretical skills in the field of trauma patient care, and the exclusion of the use of additional teaching aids by the respondents. For the level of knowledge to be higher now, it is necessary to introduce changes in the number of hours in medical studies devoted to issues related to trauma patients, where their increase should be considered. Healthcare professionals, especially those involved in emergency medicine, should be encouraged to attend certified trauma courses.

## Appendices

Questionnaire

1. Gender:  

a. Female

b. Male 

c. I decline to answer

2. Age 

a. up to 25

b. 26-35 years

c. 36-45  years 

d. 46-55  years

e. 56-65 years

f. 66 years and older

3. Education:

a. Medical student before emergency medicine classes

b. Medical student after emergency medicine classes

c. Student of emergency medical services

d. Nursing student

e. Intern

f. Emergency medicine resident

g. Surgical resident

h. Non-surgical resident

i. Emergency medicine specialist

j. Surgical specialist

k. Non-surgical specialist

l. Paramedic

m. Emergency nurse

n. Anesthesia and intensive care nurse

o. Nurse with another specialization/without specialization

4. Place of work/study:

a. City with population up to 100,000 

b. City with population from 100,000 to 250,000

c. City with population over 250,000 

5.Type of university - during education or completed:

a. Medical university

b. Private university

c. Medical course at another university

6. Participation in a certified trauma course:

a. within the past year 

b. 1-2 years ago

c. more than 2 years ago

d. never participated

7. According to ITLS guidelines, tranexamic acid in the standard management of isolated craniocerebral trauma:

a. is recommended

b. is not recommended

c. depends on the type of injury

d. I don't know this substance

8. You are called to a 15-year-old boy hit by a tram. Upon arrival at the scene, you find massive external bleeding and traumatic amputation of both lower limbs at the level of the distal femur. The patient is unconscious. He has blue lips, shallow breathing, 40 breaths/min. The chest is asymmetrical, not rising on the left side. The priority of your action upon arriving at the scene is:

a. stopping life-threatening bleeding according to the CABC scheme

b. non-instrumental airway management according to the ABC scheme

c. instrumental airway management according to the ABC scheme

d. proper securing of lost body parts

9. The medical rescue team was called to a patient in cardiac arrest. How can a paramedic independently secure the patient's airway according to current regulations?

a. cannot perform endotracheal intubation; can only use supraglottic airway devices (SGAD)

b. can perform endotracheal intubation independently after consulting with the regional medical rescue coordinator

c. can perform endotracheal intubation with muscle relaxants only after prior consultation with an emergency medicine physician

d. can perform endotracheal intubation independently

10. You arrive with the team at the scene of a traumatic cardiac arrest - the injured person fell from a 10-meter scaffolding at a construction site. Currently, CPR is being performed by other workers. According to ERC 2021 guidelines, what actions are a priority in this situation?

a. high-quality chest compressions and early defibrillation

b. the fastest possible cardiopulmonary resuscitation with simultaneous treatment of reversible causes of cardiac arrest, transport to the hospital after ROSC and stabilization of the patient's condition

c. performing critical interventions (stopping massive external bleeding, securing the airway, decompressing tension pneumothorax, cardiac tamponade), continuing CPR, and transporting the patient to the ER if mechanical chest compression is possible

d. immediate transport to the hospital for surgical treatment

11. You were called to a traffic accident. At the scene, there is a victim with an abdominal wound with evisceration. How will you secure this wound?

a. gently push the intestines back into the abdominal cavity, then disinfect and tightly dress the wound with a sterile dressing 

b. gently push the intestines into the abdominal cavity, moisten a sterile gauze with saline, and wrap the wound tightly with foil or bandage 

c. moisten a sterile dressing with saline and place it on the wound (leaving the eviscerated organs in the current state), then secure it with sterile foil 

d. lean the wound with octenidine, wrap tightly with sterile foil, and then wrap firmly with a bandage

12. Currently, according to PHTLS guidelines, the decompression of tension pneumothorax is recommended in:

a. 5th intercostal space in the anterior axillary line, secondly in the 2nd intercostal space in the midclavicular line

b. 2nd intercostal space in the midclavicular line, alternatively in the 5th intercostal space in the anterior axillary line

c. only in the 5th or 4th intercostal space in the anterior axillary line

d. only in the 2nd intercostal space in the midclavicular line

13. You were called to a traffic accident where a car collided with a truck. The truck driver was not injured. In the car were two adults and a 5-year-old child. The woman in the front passenger seat died on the spot (decapitation). The child has stable circulation and respiration and is unconscious with signs of head trauma. The driver is in shock, not logically responsive, with stable circulation and respiration, and only superficial abrasions of the limbs in the initial trauma assessment. What management of the injured should be implemented?

a. the child should be prioritized for transport to the pediatric trauma center, the driver should stay in the ambulance to calm down, have superficial injuries treated, then go to the GP from the trauma center

b. both the child and the driver should be treated as potentially severely injured patients due to the mechanism of the accident and the death of another passenger in the vehicle, and transported to the hospital as soon as possible after necessary interventions at the scene

c. both patients should be thoroughly examined in the ambulance to decide on the need for hospital transport and its level of reference

d. the passenger without vital signs, with secured airways, has the highest priority in the presence of one medical rescue team  

14. You were called to a 5-year-old girl who fell from a first-floor window. After an initial examination, it turned out that the child is in shock (no palpable peripheral pulse, unconscious, capillary refill time of 5 seconds). Two attempts were made to establish intravenous access, which did not take longer than 90 seconds. The travel time to the nearest Pediatric Trauma Center is 30 minutes. What will be the next course of action in this situation?

a. make another attempt to establish intravenous access in the other upper limb

b. give the child even a small amount of fluids orally

c. establish intraosseous access

d. abandon establishing access and transport the child to the hospital as soon as possible

15. According to ITLS guidelines, in a patient with penetrating chest trauma, dilated pupils not reacting to light, no breathing or pulse detected, and ventricular fibrillation (VF) on the ECG:

a. consider terminating cardiopulmonary resuscitation (CPR)

b. temporarily stop chest compressions and dress the wound that is the source of bleeding

c. increase the frequency of rescue breaths because the lack of pupil reaction to light is caused by brain hypoxia

d. perform defibrillation and then continue cardiopulmonary resuscitation (CPR)

16. If the injured person has a foreign body embedded in the precordial area of the chest and goes into cardiac arrest, you should:

a. immediately start cardiopulmonary resuscitation (CPR), even at the cost of ineffective chest compressions

b. remove the foreign body if it prevents effective chest compressions and start cardiopulmonary resuscitation (CPR)

c. abandon cardiopulmonary resuscitation (CPR)

d. stabilize the foreign body and then start cardiopulmonary resuscitation (CPR) by compressing the upper abdomen

17. You were called to an accident involving a motorcyclist. Upon arrival at the scene, it turned out that the injured person had lost consciousness a few minutes earlier. Witnesses were afraid to provide assistance. You recognize cardiac arrest in the man. What should be done with the helmet the motorcyclist is wearing?

a. leave the helmet because it stabilizes the cervical spine

b. quickly remove the helmet alone, minimizing the head movement of the injured person

c. remove the helmet as follows: one person stabilizes the cervical spine of the injured person, and the other removes the helmet with side-to-side movements

d. remove the helmet as follows: one person stabilizes the cervical spine of the injured person, and the other removes the helmet with up-and-down movements

18. Which procedure from the following is a priority in the case of an injured person being electrocuted?

a. cervical spine stabilization

b. combined pain pharmacotherapy, e.g., paracetamol and fentanyl

c. cooling and dressing the burns using hydrogel dressings

d. placing the injured person on a long spine board and transporting them to the hospital as soon as possible

19. For which of the following patients would you use spinal immobilization with head stabilization, side blocks, straps, and a long spine board for transportation to the ambulance?

a. a 52-year-old obese woman who was involved in a high-speed collision. She claims that everything hurts. She walks independently.

b. a 45-year-old man who was stabbed in the chest during an argument with his wife and then fell down the stairs, hitting his head. He was found to have tenderness on palpation of the occiput, multi-level spinal pain, and penetrating chest injuries.

c. a 68-year-old man who fell from the last three rungs of a ladder. He claims that during the attempt to clean the gutter, he experienced dizziness. He coughs and complains of shortness of breath. You notice distended neck veins and swelling of the lower limbs.

d. a 13-year-old boy who was hit in the head with a soccer ball during a game. The force of the blow caused him to fall to the ground. The injured person complains of a headache. The nose is blue and tender.

20. According to International Trauma Life Support (ITLS) guidelines, cervical collars:

a. should not be used in isolated penetrating chest injuries. 

b. in patients with cardiac arrest suspected of spinal injury - before starting cardiopulmonary resuscitation - a cervical collar is applied 

c. the necessity of their use should be considered in conscious patients without signs of focal injuries or neurological symptoms

d. A and C are true

21. Which statements about pelvic injuries are true (based on ITLS guidelines)?

1. The pelvis should not be squeezed too hard.

2. In the case of pelvic injuries, we must be aware of the development of hemorrhagic shock.

3. If we have an unstable pelvis, we examine it only once.

4. A suspected pelvic fracture is treated with stabilization using a pelvic belt or scarf.

a. 2, 3, 4

b. 2,  4

c. 1, 2, 3, 4

d. 1, 2, 4

22. FAST Examination:

1. It is useful in accelerating the diagnosis of massive intra-abdominal bleeding in cases of blunt abdominal trauma.

2. We examine the following organs using ultrasound: liver, spleen, kidneys, heart, lesser pelvis, and urinary bladder.

3. Examination for the presence of signs of massive internal bleeding into the abdominal cavity and pericardium.

4. It can only be performed by a specialist in emergency medicine. 

True:

a. 1, 2, 3, 4 

b. 1, 2, 3 

c. 1, 3 

d. 1, 2

23. When should you stop intravenous fluid administration to a patient in hemorrhagic shock?

a. when the patient spontaneously opens their eyes. 

b. when the patient's peripheral pulse is palpable. 

c. when we achieve the norm of arterial blood pressure and heart rate.

d. none of the above.

24. When would you consider hyperventilation in an intubated patient with an isolated head injury (according to ITLS)?

a. in every patient with a GCS <8 

b. in every patient with anisocoria 

c. only in patients with signs of brain herniation syndrome after correcting hypotension and hypoxia T

d. there are no indications for such a treatment strategy

25. To prevent post-traumatic hypothermia, what temperature should you maintain in the medical compartment of an ambulance?

a. ≥29°C

b. ≥23°C

c. ≥21°C

d. ≥18°C

26. What fluid therapy scheme will you use for a burned patient with over 20% of body surface area burned?

a. within 24 hours, administer 2-4 ml of lactated Ringer's solution × kg body weight × % of burned body surface area

b. within 24 hours, administer 4-8 ml of lactated Ringer's solution × kg body weight × % of burned body surface area

c. within 24 hours, administer 2-4 ml of 0.9% NaCl × kg body weight × % of burned body surface area

d. within 24 hours, administer 4-8 ml of 0.9% NaCl × kg body weight × % of burned body surface area

27. In a traffic accident, a 33-year-old woman lost her lower limb - traumatic amputation. A witness to the incident applied a tactical tourniquet 6 cm above the bleeding site. Unfortunately, bleeding from the wound persists. The woman is breathing spontaneously and is conscious. According to the latest TCCC recommendations, what would you do first at the scene?

a. release the tactical tourniquet and apply it again

b. apply a second tactical tourniquet above the first one

c. apply sterile gauze and a pressure bandage

d. administer hemostatic drugs to the patient

28. A 57-year-old man, after a motorcycle accident. EMS at the scene found the man conscious, breathing normally, BP 112/82 mmHg, HR 112/min, maintaining verbal contact, and logical. In the trauma examination, a closed forearm fracture was found. The X-ray in the ER confirms the initial diagnosis. The fracture was stabilized. What should you do after stabilizing the fracture?

a. check the pulse on the fractured limb distally

b. check the pulse comparatively on both limbs (using a pulse oximeter for support)

c. check the pulse comparatively on both limbs (using a pulse oximeter for support) and sensation on the fractured limb

d. do not check the pulse

29. EMS is called to a conscious man who fell from a chair at home. At the scene, the patient is calm, vital signs are stable, and eFAST is negative. EMS, in the trauma examination, found a closed femoral fracture. The patient reports pain on the NRS scale of 9/10. It was decided to administer opioids (fentanyl) as part of pain management. Will you administer fentanyl?

a. at the maximum allowable dose 

b. only if 15 minutes after administering NSAIDs, effective analgesia is not achieved

c. by titration, until a satisfactory therapeutic effect is achieved, assessing consciousness before administration and with each subsequent dose 

d. I will not administer fentanyl, as it is prohibited outside the hospital/I do not have the authority

30. You approach a patient after a traffic accident. The patient is conscious, answers your questions illogically, and does not know where they are or what day it is. What is the AVPU score?

a. A

b. V

c. P

d. the AVPU scale is not used to assess consciousness

31. Beck's triad does not include:

a. excessive filling of the external jugular vein

b. hypotension 

c. muffled heart sounds 

d. bradycardia

32. Which of the following is not part of the pediatric assessment triangle after trauma (according to ITLS)?

a. skin color

b. assessment of respiratory effort

c. circulation assessment 

d. appearance

33. Sellick's maneuver - choose a false sentence:

a. According to ITLS, it is recommended.

b. Sellick's maneuver causes closure of the esophagus, as well as lowering of the larynx and better visualization of it.

c. However, too much pressure can make ventilation and intubation difficult for the patient.

d. It is a technique that facilitates endotracheal intubation and prevents vomiting during its performance.

34. According to PHTLS guidelines:

a. despite normal cerebral perfusion, fluid administration is necessary 

b. crystalloid should be heated to 39°C 

c. do not recommend using intraosseous access to the humeral head 

d. the coexistence of internal bleeding with a head injury is an absolute contraindication to the administration of TXA

35. According to ITLS, indicate the true sentence about tranexamic acid (TXA):

a. ITLS recommends the use of TXA in patients with isolated head injury 

b. TXA is most effective when administered within 12 hours of injury

c. ITLS supports the use of TXA in patients with symptoms of hemorrhagic shock after injury within the range specified by the medical supervision system and local procedures.

d. TXA cannot be combined with initial fluid therapy and control of external bleeding

36. When transporting pregnant women, in the absence of contraindications and suspicion of injury, it is recommended to:

1. Lift the right hip by 10-15 cm, place a rolled towel underneath, and manually move the pregnant uterus to the left side.

2. Tilt the long spine board to the right side at an angle of 5-10°. 

3. Tilt the long spine board to the left side at an angle of 15-30°.

4. Lift the left hip by 10-15cm, place a rolled towel underneath, and manually move the pregnant uterus to the left side.

a. 1,3

b. 1,2

c. 2,3

d. 1,4

37. You are treating a patient with a traumatic brain injury (TBI), with developing epidural hematoma. CT scan reveals a left-sided epidural hematoma measuring 25mm. Indicate the typical symptoms of epidural hematoma:

a. decreased blood pressure, tachycardia, irregular breathing 

b. increased blood pressure, tachycardia, irregular breathing 

c. decreased blood pressure, bradycardia, irregular breathing

d. increased blood pressure, bradycardia, irregular breathing

38. You have been called to a patient with suspected traumatic brain injury (TBI) (fluid leaking from the ear, Battle's sign). You observed: no massive external bleeding, clear airways, normal vital signs, and a noticeable smell of alcohol. The patient is aroused, behaving aggressively, and expressing profanities towards you. What steps are recommended in such a situation?

a. administer sedative drugs and promptly transport to the appropriate emergency department

b. call the police to the scene to transfer the patient to a detoxification room

c. you note a lack of consent for treatment and recommend continuing treatment after obtaining consent

d. administering calming medication in suspected TBI is not recommended

39. A 43-year-old patient lost a finger and part of the hand due to a traffic accident (severed as if by a guillotine by metal). The stump was dressed with a sterile dressing and wrapped with an elastic bandage. The amputated limb part was found at the accident scene. To increase the potential success of reimplantation, the amputated body part should be:

a. rinse with 0.9% NaCl, wrap with sterile gauze, and place in a plastic bag, then store in a cooled container

b. wrap with sterile gauze and place in a plastic bag

c. place in a cooled 0.9% NaCl solution

d. place in a plastic bag with ice cubes

40. Which of the following splints allows for immobilizing both extremities of a patient if necessary?

a. Thomas splint 

b. Johnson splint

c. Here splint 

d. Sager splint

41. In Poland, there are:

a. 17 Trauma Centers and 8 Pediatric Trauma Centers 

b. 16 Trauma Centers and 16 Pediatric Trauma Centers 

c. 12 Trauma Centers and 4 Pediatric Trauma Centers 

d. 16 Trauma Centers and no Pediatric Trauma Center

42. A 27-year-old man was swimming in a lake after consuming alcohol. At one point, he began to drown. Bystanders managed to pull him ashore. The victim is not breathing, and there is no pulse palpable on the carotid artery. What will be the initial rescue procedure in this case? 

a. 30 chest compressions, then 2 breaths

b. 2 breaths, then 30 chest compressions 

c. 5 breaths, then 30 chest compressions 

d. 30 chest compressions, then 5 breaths 

43. Which of the following actions should be performed first when providing assistance to a person injured by electricity?

a. disconnect the power source

b. treat any burns 

c. transport to the hospital as quickly as possible 

d. initiate cardiopulmonary resuscitation (CPR) 

44. How should you initially proceed in the case of choking a conscious adult?

a. lean the person forward, then deliver five back blows

b. embrace the victim from behind, clasping hands into a fist, and pull forcefully inwards and upwards 

c. lean the person forward, then deliver five abdominal thrusts 

d. instruct the person to raise their arms upwards

45. In what situation can you interrupt rapid trauma assessment?

a. when sudden cardiac arrest occurs

b. when there is massive external bleeding that cannot be controlled by pressure 

c. when there is a threat at the scene of the incident

d.all of the above

46. Using a self-inflating bag with a large oxygen reservoir allows for achieving FiO2 at a level of:

a. 0,21 (21%)

b. 0,6 (60%)

c. close to (100%)

d. up to 0,8 (80%)

47. What is the correct ratio of chest compressions to rescue breaths in adult CPR?

a. 30:4

b. 30:2 

c. 15:2 

d. 30:1 
